# Does the message matter: A strategy to improve retention in recruitment registries

**DOI:** 10.1002/alz.091840

**Published:** 2025-01-09

**Authors:** Yongjun Lee, Joshua D Grill, Daniel L Gillen, Megan G. Witbracht, Dan Hoang, Brandon Delehoy

**Affiliations:** ^1^ University of California, Irvine, Irvine, CA USA; ^2^ Institute for Memory Impairments and Neurological Disorders, University of California, Irvine, Irvine, CA USA

## Abstract

**Background:**

To be successful, recruitment registries must retain participants until they can be recruited to studies. Yet, strategies and tactics to improve retention in online recruitment registries remain understudied. This study seeks to quantify the preferences of registry participants’ when it comes to video message content aimed at increasing registry renewal.

**Method:**

Active participants of the UC Irvine Consent‐2‐Contact (C2C) Registry were invited to participate in a discrete choice experiment to test whether four short video messages about the reasons to renew, delivered alone or in combination, would elicit a clear preference. The messages highlighted reasons that individuals might choose to renew their enrollment in the registry: 1) some recruiting studies offer financial compensation, 2) to advance research toward improved outcomes in health and disease, 3) to be matched to a study relevant to one’s own health, and 4) promotion of novel research within the registry using data collected at renewal. From 15 possible combinations, participants viewed 7 random pairs of videos. After viewing each pair, participants were asked to select the video that would most compel them and others to renew their participation in the registry. Each participant was asked to complete a final survey including 8 Likert scale items assessing participant attitudes toward renewal. The study employed a Bradley‐Terry model to identify the combination deemed favored by the participants. To estimate topic‐specific preferences, interaction between the topics across all combinations, along with covariate adjustments for participant demographics were modeled via an extension of the Bradley‐Terry model in combination with the study’s fractional factorial design.

**Result:**

Statistical power to identify the most preferred topic along with topic‐specific orderings were carried out via simulation. Data collection is underway. Presented results will include efficacy of individual messages as well as optimal message combinations and concordance with survey results.

**Conclusion:**

This study introduces a novel design where the effect of video messages from a fractional factorial combination of four incentives can be estimated through a paired rank analysis model. Results will inform a future randomized factorial design trial of retention interventions.

